# Genotypic and Phenotypic Characterization of MRSA Colonization and Persistence in Residential Aged Care Facilities in Adelaide, Australia

**DOI:** 10.1155/ijm/4145293

**Published:** 2026-10-19

**Authors:** Belaynesh T. Asrat, Sylvia A. Sapula, Naomi L. Siderius, Jonathan J. Whittall, Anteneh Amsalu, Bradley J. Hart, Manish Kumar, Morgyn S. Warner, John Turnidge, Henrietta Venter

**Affiliations:** ^1^ School of Pharmacy and Biomedical Science College of Health Adelaide University Adelaide South Australia Australia adelaide.edu.au; ^2^ Department of Medical Microbiology University of Gondar Gondar Ethiopia uog.edu.et; ^3^ Microbiology and Infectious Diseases SA Pathology Adelaide South Australia Australia sapathology.sa.gov.au; ^4^ School of Medicine College of Health Adelaide University Adelaide South Australia Australia adelaide.edu.au; ^5^ Infectious Diseases Unit Royal Adelaide Hospital Adelaide South Australia Australia rah.sa.gov.au; ^6^ School of Biological Sciences College of Science Adelaide University Adelaide South Australia Australia adelaide.edu.au

**Keywords:** antimicrobial resistance, clonal dissemination, genotypic–phenotypic comparison, hospital-acquired MRSA, MRSA carriage, multidrug resistance

## Abstract

**Background:**

Residential aged care facilities (RACFs) are potential reservoirs for methicillin‐resistant *Staphylococcus aureus* (MRSA), with residents at increased risk due to compromised immunity, comorbidities, frequent hospital visits, and close living quarters.

**Objectives:**

This study is aimed at assessing MRSA prevalence in RACFs and analyzing antimicrobial resistance mechanisms both phenotypically and genotypically.

**Methods:**

A total of 106 participants were recruited: 84 from three RACFs and 22 from retirement villages (as control samples). MRSA was isolated from nasal, wound, and urine samples using selective media. Identification was confirmed via MALDI‐TOF MS. Antimicrobial susceptibility testing and MRSA screening were conducted using ISO broth microdilution and EUCAST disc diffusion. Whole‐genome sequencing and bioinformatics were used to explore resistance mechanisms and clonal transmission.

**Results:**

Nasal carriage of MRSA was 8.2% (*n* = 7/85) among RACF residents; none were found in retirement village participants. All isolates formed strong biofilms, and 66.6% (10/15) showed multidrug resistance. Genotypic analysis revealed ST22 as the predominant clone. Genotypic resistance largely matched phenotypic profiles, except for unexplained tetracycline resistance. No isolates carried Panton–Valentine leukocidin (PVL), consistent with previously reported data on MRSA in long‐term care facilities. SNP‐based phylogenetic analysis indicated persistent colonization and circulation of multidrug‐resistant clones within the facilities. Nasal carriage of MRSA was associated with UTIs and/or wound infections in some cases, whereas a UTI caused by the same clone in a resident within the same facility without nasal carriage indicates possible intrafacility clonal dissemination.

**Conclusion:**

This study highlights persistent colonization with hospital‐associated MRSA and potential clonal transmission among residents of aged care facilities despite several courses of antibiotics. The predominance of the epidemic ST22 clone and high rates of multidrug resistance underscore the urgent need for targeted infection control, routine genomic surveillance, and antimicrobial stewardship in long‐term care settings to prevent MRSA dissemination.

**Impact Statement**

Limited data exist on the prevalence, genomic characteristics, and colonisation and persistence of methicillin‐resistant *Staphylococcus aureus* (MRSA) in residential aged care facilities (RACFs).

## 1. Introduction

MRSA is a major nosocomial pathogen and a global therapeutic challenge [1]. It is primarily linked to skin and soft tissue infections [2], whereas MRSA bacteremia carries high mortality rates (15%–60%) [3], prolonged hospital stays, and increased healthcare costs [4]. In 2021, MRSA caused an estimated 130,000 deaths [5].

MRSA infections are particularly concerning in the elderly and in healthcare settings [6], including RACFs [7]. Global MRSA colonization in long‐term care residents is estimated at 14.69% [8], with a 16% prevalence reported in Melbourne RACFs [9]. Between 2015 and 2023, MRSA prevalence in RACFs surpassed that in hospitals and community settings across Australia [7]. In addition to MRSA, an increased prevalence of other antimicrobial resistant bacteria has also been reported in RACFs [10–12]. Contributing factors include compromised immunity, comorbidities, indwelling devices, and high antibiotic use—often without documented infection [9, 13]. In RACFs in Australia, 65% of antimicrobials were prescribed without symptoms, and 37.6% were used for over 6 months [14, 15]. Biocide overuse and frequent hospital transfers further increase exposure to resistant pathogens [16].

Methicillin resistance arises from the staphylococcal cassette chromosome *mec* (SCC*mec*), which carries the *mecA* gene encoding PBP2a, conferring resistance to nearly all *β*‐lactam antibiotics [17]. SCC*mec* may also carry resistance genes for other antibiotics, whereas efflux pumps and mutations contribute further resistance [18–20].

Despite its public health impact, recent data on MRSA in RACFs remain limited. This study conducted 1‐year surveillance in three Adelaide RACFs and retirement villages, isolating MRSA from nasal swabs, urine, and wounds. Phenotypic and genotypic characterization, including SNP analysis, was used to investigate colonization and clonal transmission within and between facilities.

## 2. Methods

### 2.1. Sampling

Nasal swabs, urine, and wound samples were collected from participating residents residing in three RACFs (“Facility A,” 170 beds; “Facility B,” 70 beds; “Facility C,” 58 beds) and an independent‐living retirement facility for residents that do not need ongoing care (“Retirement,” individual houses in the same location as the retirement facilities) in Adelaide, Australia. The average age of the RACF residents was 87 years with a ratio of 1:1.76 male to female RACF participants. All samples were collected on‐site by a registered nurse between October 2019 and February 2021. All samples were kept on ice and transported for processing on the day of collection.

### 2.2. Isolation and Identification of MRSA

Nasal, urine, and wound swabs were streaked on to Brilliance MRSA plate (Thermo Fisher, Scientific, Australia), BrillianceUTI plate (OxoidTM, Australia), and blood agar plates (Sigma Aldrich), respectively. Following incubation at 37°C for 16–24 h, a single colony of presumptive *S*. *aureus* was selected from each plate [21] and processed for identification using matrix‐assisted laser desorption/ionization time‐of‐flight mass spectrometry (MALDI‐TOF) (Bruker Daltonik GmbH Bremen, Germany) in the Australian Centre for Antimicrobial Resistance Ecology. Confirmed *S*. *aureus* isolates were stored at −80°C in Tryptone Soy broth (CM0129, Oxoid) supplemented with 20% (v/v) glycerol.

Further confirmation of MRSA isolates was conducted by using cefoxitin disc diffusion carried out according to European Committee on Antimicrobial Susceptibility Testing (EUCAST) recommendations [22]. An inhibition zone smaller than the established threshold (21 mm) was considered indicative of MRSA [23].

### 2.3. Antimicrobial Susceptibility Testing Investigates the Multidrug Resistance (MDR) Status of MRSA Isolates

To investigate the resistance pattern of MRSA isolates to other antibiotic classes and biocides, minimum inhibitory concentration (MIC) was determined using the broth dilution method according to ISO 20776‐1 as prescribed by EUCAST [24] and described previously [11]. *S*. *aureus* ATCC 25923 was used as a quality control. Isolates were classified as resistant and susceptible to different classes of antibiotic according to the 2025 EUCAST break point [23]. Since neither breakpoint nor epidemiological cutoff (ECOFF) values for biocides are available from any standards setting organization, estimated wild‐type cutoff values from Morrissey et al. [25] were used to classify isolates as wild‐type and nonwild‐type for biocides. MRSA isolates were classified as MDR if they demonstrated resistance to at least one agent in each of three antimicrobial classes, excluding methicillin resistance [26].

Inducible clindamycin resistance was assessed with the “D zone” test [27]. Briefly, an erythromycin (15 *μ*g) disc was placed 15 mm (edge to edge) from a clindamycin (2 *μ*g) disc on a Mueller–Hinton agar plate, previously inoculated with a 0.5 McFarland MRSA suspension. Following overnight incubation at 37°C, the appearance of a flattened (D‐shaped) zone around clindamycin between the two discs was indicative of inducible clindamycin resistance.

### 2.4. A Checkerboard Assay to Evaluate Efflux Pump Inhibition

To evaluate the effect of efflux pump inhibitors on the MIC values, checkerboard assays were performed as described previously [28]. In short, bacterial cultures were allowed to grow in a medium containing varying concentrations of the antimicrobial compound in the absence or presence of the selected efflux pump inhibitor omeprazole [29]. Omeprazole was used at a concentration ranging from 0 to 128 mg/L, with twofold serial dilutions. The cultures were incubated for 18 h, and growth was evaluated by measuring OD_600_ using a Thermosteric Multiskan FC photometer (Thermofisher, Australia). Checkerboard assays were conducted in duplicate.

### 2.5. Evaluation of MRSA Biofilm Mass Quantification

The ability of MRSA isolates to form a biofilm was assessed with crystal violet using round‐bottomed 96‐well microtiter polystyrene plates (Corning, Victoria, Australia) [30]. Biofilm growth was measured at OD_620_ nm using micro plate reader by using Thermo scientific Multiskan (Fisher Scientific). The optical density of each sample (OD_s_) was used as an indicator of biofilm formation and compared with the critical OD of the control (ODc) to differentiate biofilm‐forming ability.

The OD_c_ was determined by averaging the absorbance of negative controls. Biofilm production was categorized as follows: OD_s_ ≤ 2 × OD_c_ indicated weak biofilm production, 2 × OD_c_ < OD_s_ < 4 × OD_c_ indicated moderate biofilm production, and OD_s_ ≥ 4 × OD_c_ indicated high biofilm production [31–33]. *S*. *aureus* ATCC 25923, a known biofilm producer [34, 35] and *S*. *epidermidis* ATCC 12228 [36] were used as a positive and negative control, respectively.

### 2.6. Genomic Extraction for Whole Gene Sequencing

Genomic DNA for whole‐genome sequencing (WGS) was extracted using the MN NucleoSpinMicrobial DNA kit (Machery‐Nagel GmbH and Co.KG, Duren, Germany), following the manufacturer′s instructions. The quantity (ng/*μ*L) and quality (absorbance 260:280 nm) of extracted DNA was assessed using a Cytation5 imaging reader (BioTek instruments, Winooski Vermont, United States). Purified genomic DNA was visualized on 1% w/w agarose gel electrophoresis to ensure no shearing of extracts and was stored at −20°C.

WGS was performed at SA Pathology (Adelaide, Australia) using the Illumina NextSeq 550 platform. Sequencing library preparation was carried out using the Nextera XT DNA library preparation kit (Illumina Inc., United States) as per manufacturer′s instructions. Sequencing was performed on the Illumina NextSeq 550 platform with the NextSq 500/550 Mid‐Output kit v2.5 for 300 cycles (Illumina Inc.).

### 2.7. Bioinformatic Analyses

Raw paired‐end sequenced reads (150 bp) were assembled and annotated using the TORMES v.1.3 pipeline. AMR genes were screened using, ResFinder [37], ARG‐ANNOT [38], and the comprehensive antimicrobial resistance database (CARD) [39]. Chromosomal point mutations were assessed using CLUSTAL OMEGA and the Point Finder database [40]. Additionally, plasmids were detected using ABRicate [41], with Plasmid Finder being employed for identification of plasmids [42]. The search of virulence genes was performed by screening each genome against the Virulence Factors Database (VFDB) [43]. The SCC*mec* (staphylococcus cassette chromosomes *mec*) typing of MRSA isolates was performed using the SCCmecFinder database tools (https://cge.food.dtu.dk/services/SCCmecFinder).

A phylogenetic tree was constructed based on core genome SNPs using CSI phylogeny v.1.4 (https://cge.cbs.dtu.dk/services/CSIPhylogeny) [44]. *S*. *aureus* NZ‐KU510528.1 (representing ST22) was used as a reference genome for SNP analysis. The final phylogenetic tree was visualized using the Interactive Tree of Life (iTOL v.6.0) [45]. A core genome‐based phylogeny was constructed for all analyzed isolates, indicating a predominant core genome in the WGS data, which is primarily used for SNP analysis. SNP analysis was employed to observe clonality within isolate clusters.

### 2.8. Statistical Analysis

Statistical analysis of the biofilm values for each isolate compared with the negative control was conducted using a one‐way ANOVA test in GraphPad Prism 10.2. Bar graphs illustrating the results were generated with Graph Prism v10.2.

## 3. Results

### 3.1. Prevalence and Persistence of MRSA Nasal Colonization in RACF Residents

A total of 198 nasal swabs, 42 urine samples, and 11 wound samples were collected from 85 residents across three RACFs, along with 44 nasal swabs and 3 urine samples from 29 residents living in retirement villages. To assess persistent colonization, nasal swabs were collected in two rounds. Of the 114 individuals sampled, 68 RACF residents provided two nasal swabs, collected 3–6 months apart. The remaining RACF individuals provided one nasal sample only, as part of the first collection, with certain individuals leaving the aged care facilities or withdrawing from the study.

All nasal swabs and *S*. *aureus* isolates recovered from urine and wound samples were screened on Brilliance MRSA selective agar for the presumptive identification of MRSA. This screening resulted in a total of 15 MRSA isolates recovered from nine (*n* = 9/85, 10.5%) residents residing in three RACFs, whereas no MRSA isolates were recovered from retirement village residents (Table 1). Of the nine MRSA positive individuals, seven (*n* = 7/85, 8.2%) returned positive nasal swabs and two had MRSA positive urine samples. Of the 15 isolates recovered from RACFs residents, 10 were recovered from nasal swabs (66.6%), four from urine (26.6%) and one from a wound sample (6.6%). Ten MRSA were isolated from Facility 1, one from Facility 2, and four from Facility 3.

**Table 1:** Number and type of samples collected along with the distribution of the 15 MRSA isolated by facility and sample types.

Facility	Specimens						
		Nasal swabs^a^		Urine		Wound	
	Individuals sampled (*n*)	Samples (*n*)	MRSA detected *n*(%)	Samples (*n*)	MRSA detected *n*(%)	Samples (*n*)	MRSA detected *n*(%)
Facility‐1	42	77	6 (7.8)	22	3 (13.6)	8	1(12.5)
Facility‐2	28	49	0	7	1 (14.2)	1	0
Facility‐3	15	28	4 (14.2)	13	0	2	0
RV	29	44	0	3	0	0	0

Abbreviations: %, percentage; *n*, number of samples collected; N/A, not applicable as urine and wound samples were collected upon presentation of UTI symptoms or presence of wounds, respectively; RV, retirement village.

^a^Not all residents were available or consented to sampling in Round 2.

### 3.2. Antimicrobial Resistance Profile of MRSA Isolates

The presumptive MRSA was confirmed as *S*. *aureus* with MALDI‐TOF MS and as MRSA with disc diffusion using the cefoxitin screening test [22]. All the isolates, apart from SA2481, were cefoxitin resistant and hence confirmed as MRSA (data not shown).

As expected, broth dilution reveals that all the isolates were resistant to cefoxitin with MIC values of ≥ 64 mg/L, whereas SA2481 was borderline cefoxitin resistant with an MIC of 16 mg/L (Figure 1). Furthermore, four (26.6%) of these isolates displayed high levels of cefoxitin resistance with MIC values of ≥ 256 mg/L being observed. High levels of resistance were also observed against piperacillin–tazobactam, with MICs of ≥ 64 mg/L for 93% (*n* = 14/15) of isolates. In addition, 80% (*n* = 12/15) and 53.3% (*n* = 8/15) of isolates were ciprofloxacin and erythromycin resistant, respectively. Finally, 40% (*n* = 6/15) of isolates displayed low level (MIC values of 2 mg/L) daptomycin resistance, whereas two isolates displayed low level resistance against the broad‐spectrum antibiotics, tetracycline, and doxycycline. One isolate (SA3499) displayed low level tigecycline resistance with an MIC of 4 mg/L. None of the isolates were resistant to mupirocin or clindamycin (Figure 1); however, all the erythromycin resistant isolates displayed inducible clindamycin resistance (results not shown).

**Figure 1:**
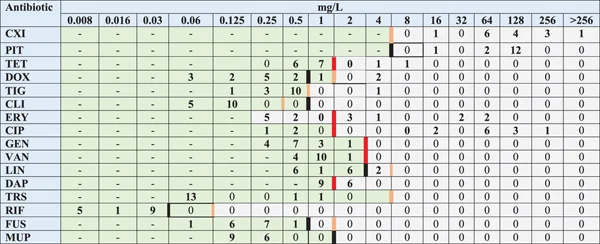
Minimum inhibitory concentration (MIC) distribution frequencies of MRSA isolates obtained from RACFs. Vertical black lines indicate EUCAST ECOFF values, whereas the orange lines indicate the clinical breakpoints according to EUCAST, and the red line indicates antimicrobials that have the same ECOFF and breakpoint. MICs of isolates greater than the ECOFF values are presented in the next highest dilution in the no shade region and reported as greater than the MIC value, whereas green shaded region indicates the MIC values that were less than the breakpoints. CXI, cefoxitin; PIT, piperacillin–tazobactam; TET, tetracycline; DOX, doxycycline; TIG, tigecycline; CLI, clindamycin; ERY, erythromycin; CIP, ciprofloxacin; GEN, gentamycin; VAN, vancomycin; LIN, linezolid; DAP, daptomycin; RIF, rifampicin; TRS, trimethoprim sulfamethoxazole; FUS, fusidic acid; MUP, mupirocin.

No biocide tolerance was observed for any of the MRSA isolates assessed in this study (Figure 2). The estimated wild‐type cutoff value of silver nitrate for *S*. *aureus* has not been previously established or reported. However, we observed MIC values of 8 and 16 mg/L, which are consistent with other studies reporting MIC values ranging from 16 to 32 mg/L for silver nitrate against *S*. *aureus* [46].

**Figure 2:**
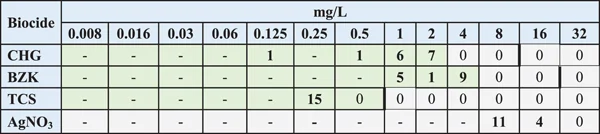
Biocide susceptibility profile for MRSA isolated from RACFs. CHG, chlorhexidine gluconate; BZK, benzalkonium chloride; TCS, triclosan; AgNO_3_, silver nitrate. The vertical black lines indicate ECOFF values as determined by Morrissey et al. [25].

Looking at individual isolates, 66.6% of the isolates are multidrug resistant (10/15), and several of these isolates displayed high‐level resistance to antimicrobials other than cefoxitin. For example, 12 isolates exhibited MIC values of 16–256 mg/L for ciprofloxacin (8–128 times the ECOFF value) (Figure 1).

Isolate SA2481, which was classified as MSSA according to the cefoxitin disc diffusion method had low level cefoxitin resistance (MIC 16 mg/L) when assessed using the microbroth dilution method. This isolate displayed low‐level resistance against piperacillin–tazobactam (MIC 16 mg/L) and was one of the four isolates displaying a high MIC value of ≥ 32 mg/L for erythromycin (Table 2).

**Table 2:** Sequence types (STs), minimum inhibitory concentrations (MICs), and EUCAST breakpoints for antimicrobials used in this study and sample type information of sequenced 15 MRSA isolates.

Facility	Isolate	MLST	Source	CXI	PIT	DOX	TIG	TET	CIP	VAN	LIN	GEN	DAP	TRS	RIF	ERY	FUS	CLI	MUP
		EUCAST Breakpoint		> 4	N/A	> 1	> 0.5	> 1	> 1	> 2	> 4	> 2	> 1	> 4	> 0.06	> 1	> 1	> 0.25	N/A
Facility 1	SAA237	6963	Nasal	256	128	0.25	1	0.5	64	0.5	4	0.25	1	0.06	< 0.03	0.25	0.125	0.0625	0.125
Facility 1	SA2415	6963	Nasal	> 256	128	0.0625	0.25	0.5	128	1	0.5	1	1	0.0625	< 0078	0.5	0.25	0.0625	0.25
Facility 1	SAA238	78	Nasal	128	128	0.25	0.5	0.5	0.25	1	2	0.25	1	0.0312	< 0.03	0.5	0.125	0.0625	0.125
Facility 1	SA5943	78	Nasal	128	64	0.25	0.5	1	0.5	2	2	2	1	1	0.0078	0.25	0.125	0.125	0.25
Facility 1	SAA0035	6963	Nasal	64	128	4	0.5	1	64	1	0.5	0.5	2	0.0625	< 0.0039	2	0.25	0.065	0.125
Facility 1	SA2148	1	Nasal	64	128	0.125	0.5	0.125	0.5	1	2	1	2	0.06	0.0078	0.25	0.0625	0.125	0.125
Facility 1	SAA519	22	Wound	64	64	0.25	1	1	64	0.5	2	0.25	1	0.0625	< 0.03125	64	0.25	0.125	0.25
Facility 1	SA2285	22	Nasal	64	64	0.5	0.25	1	64	1	1	0.5	1	0.06	< 0.0039	4	0.25	0.125	0.25
Facility 1	SA2443	22	Urine	64	128	0.0625	0.5	0.5	64	1	0.5	0.5	1	0.0625	< 0.0078	32	0.25	0.125	0.125
Facility 1	SA2467	22	Urine	128	128	0.0625	0.125	2	128	1	0.5	0.5	1	0.06	< 0.0039	2	0.25	0.125	0.25
Facility 2	SA2481	45	Urine	16	16	< 0.0625	0.25	0.5	16	1	0.5	1	2	0.5	0.0156	32	0.5	0.125	0.25
Facility 3	SAA254	22	Nasal	> 256	128	0.25	0.5	0.5	128	0.5	2	0.25	1	0.0625	0.03125	64	0.125	0.0625	0.125
Facility 3	SA6221	22	Nasal	256	128	0.125	0.5	1	256	1	4	0.5	1	0.06	< 0.0039	2	0.125	0.125	0.125
Facility 3	SAA005	22	Nasal	64	128	4	0.5	4	64	0.5	0.5	0.5	2	0.06	0.0039	0.25	0.125	0.125	0.125
Facility 3	SA3499	22	Nasal	128	128	4	4	8	128	1	2	0.5	2	0.06	< 0.0039	< 0.25	0.25	0.125	0.25

Abbreviations: CIP, ciprofloxacin; CLI, clindamycin; CXI, cefoxitin; DAP, daptomycin; DOX, doxycycline; ERY, erythromycin; FUS, fusidic acid; GEN, gentamicin; LIN, linezolid; MUP, mupirocin; PIT, piperacillin–tazobactam; RIF, rifampicin; TET, tetracycline; TIG, tigecycline; TRS, trimethoprim sulfamethoxazole; VAN, vancomycin.

### 3.3. Differences in Antibiotic Resistance and Multidrug‐Resistant Profiles Between Facilities

With many more isolates recovered from Facility 1, it is difficult to draw meaningful comparisons between facilities. However, a comparison of isolates as determined from the number of samples collected, shows a similar incidence (9.2%) of MRSA in Facilities 1 and 3. Facility 2 presented one MRSA isolate from 57 samples (1/57; 1.7%). Facility 1 has the highest prevalence of erythromycin, ciprofloxacin, and cefoxitin resistant MRSA isolates (Figure 3). In addition, doxycycline and tigecycline resistant isolates were observed only in Facility 3, with two (13.3%) and one (6.6%) isolate resistant to these compounds, respectively. Hence, Facility 1 exhibited a notable distribution of MDR isolates.

**Figure 3:**
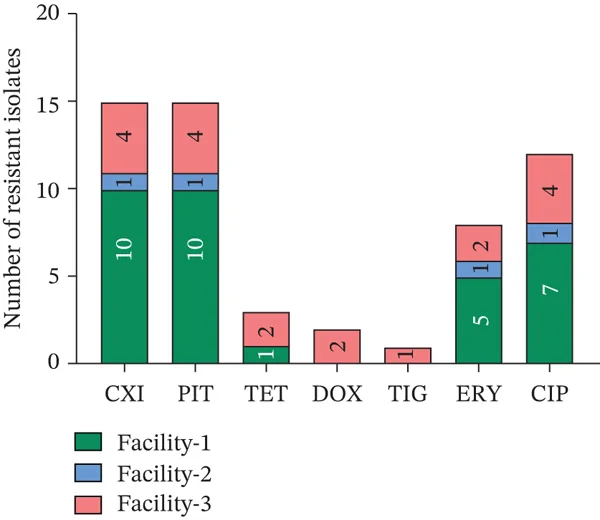
Distribution of resistant isolates for each antibiotic per facility. CXI, cefoxitin; PIT, piperacillin–tazobactam; TET, tetracycline; DOX, doxycycline; TGC, tigecycline; ERY, erythromycin; CIP, ciprofloxacin.

Further comparison of facilities revealed that Facility 1 was the only site from which MRSA (*n* = 1/15, 6.6%) resistant to four different classes of antibiotics was isolated (Figure 4A). MDR MRSA were isolated from all three facilities (Figure 4B).

**Figure 4:**
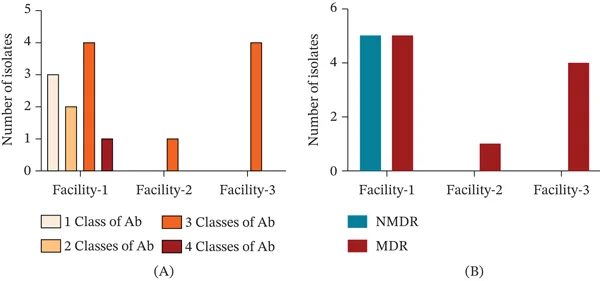
Antimicrobial resistant MRSA. (A) Total number of MRSA isolates that showed resistance to different classes of antimicrobials. (B) Distribution of MDR and non‐MDR (NMDR) MRSA isolates per facility.

### 3.4. MRSA Isolates Were Strong Biofilm Formers

As biofilm production contributes greatly to the success of MRSA to cause chronic infections [47], the production of these was assessed in this study. All 15 MRSA isolates were strong biofilm formers, with results comparable with *S*. *aureus* ATCC 25923 (Figure 5).

**Figure 5:**
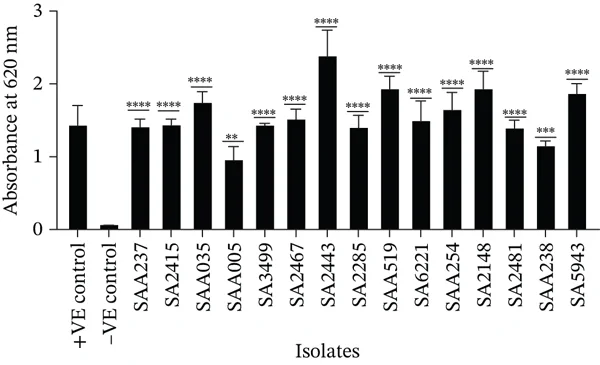
Biofilm forming ability of MRSA isolated from three RACFs in Adelaide. Biofilm production was assessed in triplicate. *S*. *aureus* ATCC 25923 (representing a strong biofilm former) and *S*. *epidermitis* ATCC 12229 were used as a positive and negative control, respectively. Bars indicate the average OD value of each isolate and control samples. The OD readings of the isolates were significantly higher than the OD value of the negative control, as tested by ordinary one‐way ANOVA (∗∗∗∗*p* < 0.0001, ∗∗∗*p* < 0.001, and ∗∗*p* < 0.01).

### 3.5. SCC*mec*, *Spa*, and Sequence Typing of MRSA Isolates

WGS was carried out on all 15 isolates, with SSC*mec* and spa (staphylococcal protein A) typing, and sequence types (STs) used to classify the isolates. Accordingly, isolates were clustered into three main SCC*mec* types, seven different spa types and five different ST (Table 3). SCC*mec*_type_IV (2B) was the predominant (*n* = 11/15; 73.3%) SCC*mec*_type followed by SCC*mec*_type_IVa (2B) (*n* = 2/15; 13.3%) and SCC*mec*_type Vc (*n* = 1/15; 6.6%).

**Table 3:** The distribution of SCC*mec* type in MRSA isolates.

Sample	SCC*mec* type^a^	Spa type	Sequence type (ST)	Clonal complex (CC)
SA2148	SCC*mec*_type_IVa 2B	t127	1	1
SAA238	SCC*mec*_type_IVa 2B	t3177	78	Unknown/novel
SA5943	SCC*mec*_type_IVa 2B	t3177	78	Unknown/novel
SA2415	SCC*mec*_type_IV 2B	Unknown/novel	6963	22
SA2467	SCC*mec*_type_IV 2B	t717	22	22
SA3499	SCC*mec*_type_IV 2B	Unknown/novel	22	22
SA6221	SCC*mec*_type_IV 2B	t608	22	22
SAA005	SCC*mec*_type_IV 2B	Unknown/novel	22	22
SAA237	SCC*mec*_type_IV 2B	t1358	6963	22
SAA254	SCC*mec*_type_IV 2B	t625	22	22
SA2285	SCC*mec*_type_IV 2B	t717	22	22
SA2443	SCC*mec*_type_IV 2B	Unknown/novel	22	22
SAA519	SCC*mec*_type_IV 2B	Unknown/novel	22	22
SAA035	SCC*mec*_type_IV 2B	Unknown/novel	6963	22
SA2481	SCC*mec*_type_Vc	t1081	45	45

^a^SCC*mec*—staphylococcus cassette chromosome *mec*.

In this study, nine isolates were analyzed to confirm the presence of seven distinct spa types. Interestingly, six of these isolates were characterized by spa types that were previously unknown or novel (Table 3). The most detected spa types among the isolates were t3177 and t717.

ST22 was the most common ST with eight of the isolates belonging to this ST (*n* = 8/15). Two isolates were ST78, whereas SA2148 and SA2481 were classified as ST1 and ST45, respectively (Table 3). SAA237, SA2415, and SA0035 were assigned to the uncommon ST6963 according to PubMLST. ST6963 and ST22 share a similar MLST profile, differing only by one nucleotide in the *gmk* housekeeping allele (ST22: *gmk* (5) and ST6963: *gmk* [552]) [48] suggesting recent divergence between ST22 and ST6963.

Isolates were further categorized into clonal complexes (CCs), where all ST22 and ST6963 isolates determined to belong to the same CC (CC22) indicating relatedness of the isolates. ST1 and ST45 isolates were classified as CC1 and CC45, respectively, whereas ST78 was unassigned to any of the CCs (Table 3).

### 3.6. *β*‐Lactam–Methicillin Resistance Determinants

In this study, *blaZ* and *mecA* genes were detected in 100% (*n* = 15/15) and 93.3% (*n* = 14/15) isolates, respectively (Figure 6). SA2481, which displayed low levels of cefoxitin resistance with MIC 16 mg/L, lacked the *mecA* and *mecC* genes. However, it possesses SCC*mec* containing the *ccr* gene complex, a hypervariable region (HVR), and the insertion sequence IS*431*, which influences the horizontal mobility and stability of SCC*mec* [49].

**Figure 6:**
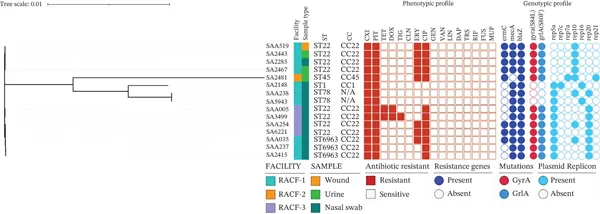
Phylogenetic SNP analysis of 15 MRSA isolated from three RACFs in South Australia. The antimicrobial susceptibility profiles are presented, and presence of AMR determinants and mutations in GyrA and GrlA/ParC are indicated. Vertical columns illustrate from left to right: facility; sample type; resistance phenotype: resistant (filled squares) and susceptible (empty squares); resistance genotype: presence (filled circles) and absence (empty circles) of resistance genes; mutations in *gyrA and grlA*/*parC*: present (filled circles) and absent (empty circles); and predominant plasmid replicon types: presence (filled circles) and absence (empty circles). CXI, cefoxitin; PIT, piperacillin–tazobactam; TET, tetracycline; DOX, doxycycline; TIG, tigecycline; CLN, clindamycin; ERY, erythromycin; CIP, ciprofloxacin; GEN, gentamicin; VAN, vancomycin; LNZ, linezolid; DAP, daptomycin; TRS, trimethoprim–sulfamethoxazole; RIF, rifampicin; FUS, fusidic acid; MUP, mupirocin.

### 3.7. Ciprofloxacin Resistance Is Associated With *gyrA*/*parC* Mutations and Efflux Pumps

Most of the MRSA isolates 80% (*n* = 12/15) were resistant to ciprofloxacin (Table 2). Mutations in the genes encoding DNA gyrase (*gyrA and gyrB*) and topoisomerase IV (*grlA*/*parC* and *grlB*/*parE*) contribute to fluoroquinolone resistance [50]. These mutations are typically located within the quinolone resistance‐determining regions of the GyrA/B and GrlA/ParC proteins [51]. In the current study, all fluoroquinolone‐resistant isolates (*n* = 12/15) carried two identical mutations, namely, Ser84Leu and Ser80Phe mutations in GyrA and GrlA/ParC, respectively (Figure 6). All the ST22 and ST6963 (CC22) isolates displayed higher levels of resistance against ciprofloxacin, with MICs of ≥ 64 mg/L. Interestingly, the borderline MRSA isolate, SA2481, contained the same mutations but displayed low‐level fluoroquinolone resistance (MIC 16 mg/L) when compared with the other isolates, indicating that additional mechanisms of ciprofloxacin resistance may be utilized by these isolates. As efflux pumps are known to export a wide range of substrates, their involvement was assessed here. To this end, antimicrobial susceptibility in the presence of omeprazole, a known inhibitor of the major facility superfamily efflux pumps [29] was assessed. Efflux‐mediated resistance was observed for four isolates: a fourfold reduction in ciprofloxacin MIC was observed for SA3499, SAA005, and SA6221 in the presence of omeprazole, whereas an eightfold reduction in ciprofloxacin MIC was observed for the low‐level ciprofloxacin resistant isolate SA2481 (isolate that lacks the *mecA* gene) (Figure 7). Increased efflux activity was also observed for these four isolates by measuring the efflux of the fluorescent substrate Hoechst 33342 in real time [52] (results not shown). Overall, these findings suggest that efflux pumps might play a role in ciprofloxacin resistance.

**Figure 7:**
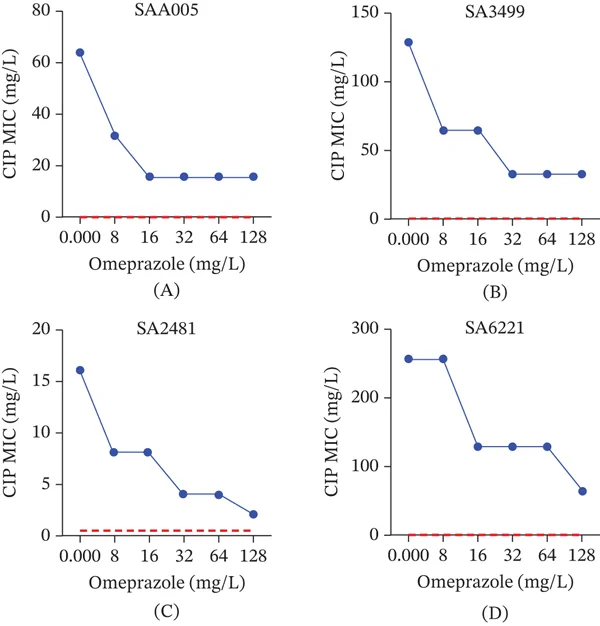
(A–D) Antimicrobial synergy assays of ciprofloxacin resistant MRSA isolates in the presence of the efflux pump inhibitor omeprazole. Data represent the susceptible MSSA ATCC25923 control strain (dotted red line) at MIC = 0.5 mg/L and the MIC of each isolate tested in the presence of omeprazole (blue line with circles). MIC, minimum inhibitory concentration; CIP, ciprofloxacin.

### 3.8. Determinants of Macrolide Resistance

Eight (*n* = 8/15, 53.3%) isolates were resistant to erythromycin and harbored the *ermC* gene (Figure 6). This gene encodes a methyltransferase that catalyzes the methylation of a specific target on the 23S rRNA, preventing antibiotics from binding to their ribosomal target [53]. Among these eight erythromycin‐resistant isolates, four displayed lower levels of resistance with MICs of 2–4 mg/L, whereas the remaining isolates exhibited high levels of resistance with MICs of 32–64 mg/L. This suggests that the presence of *ermC* alone may not be contributing to the high levels of erythromycin resistance observed. However, there is no evidence of point mutations in the ABC‐F type ribosomal protection protein MsrA (results not shown) [54].

### 3.9. Determinants for Tetracycline, Doxycycline, and Tigecycline Resistance

WGS did not reveal specific resistance mechanisms for doxycycline and tetracycline in isolate SA005 or for tigecycline, doxycycline, and tetracycline in isolate SA3499 (Figure 6). To investigate whether drug efflux pumps might contribute to the observed resistance to tetracycline, doxycycline, and tigecycline, the effects of efflux pump inhibitors, omeprazole and verapamil, were evaluated. However, the addition of these inhibitors did not reverse the resistance (results not shown), suggesting that efflux pumps may not be the primary mechanism responsible for resistance to these antibiotics.

### 3.10. Detection of Virulence Genes of the MRSA Isolates

None of the isolates carried the *lukS* and *lukF* genes, which encode the Panton–Valentine leukocidin (PVL) toxin, whereas all isolates possess five genes associated with hemolysins (*hla*, *hlb*, *hld*, *hlgA*, and *hlgB*) [55] and the genes involved in iron uptake (*srtB* and *isd*/*A*‐*G*) [56]. Additionally, genes encoding cell lysis and tissue invasion (*geh*, *lip*, *sspA*/*B*/*C*, *ebp*, *esaA*/*b*, *esxA*, and *aur*) were present in all isolates, whereas *essA*/*B was* only identified in three isolates. SA2148 carried *vwb*, a coagulase that promotes pathogenicity [57]. Enterotoxin genes (*sec*, *sell*, and *seh*) were identified in all isolates, with *seh* being the sole enterotoxin detected in SA2148. In the immune invasion cluster (*chp*, *cap8A*‐*K*, *scn*, *sak*, *sbl*, *sdrD*, and *sdrE*), all genes except *sdrD* and *sdrE* were present in all MRSA isolates. *SdrD* was found in SA3499 and SA2148, whereas *sdrE* was only detected in the SA238 isolate. The *chp* gene was detected only in ST78 isolates (SAA238 and SA5943). Most isolates possessed the adhesion and biofilm production genes (*fnbB*, *fnbA*, and *icaA*/*B*/*C*/*D*/*R*), except for *fnbA*, which was absent in ST78 (SA5943 and SAA238) and ST45 (SA2481), and *fnbB*, which was exclusively detected in the ST1 (SA2148) isolate. These results are in accordance with the biofilm analysis as all isolates were strong biofilm producers Furthermore, *lukE* and *lukD* genes, associated with pore‐forming leukotoxins, were present in SA5943, SAA238, and SA2148.

### 3.11. Prevalence of Plasmid Replicon Types Detected in MRSA Isolates

Assessment of plasmid replicons was undertaken (Table 4). The number of replicons per isolate varied between one and three. The most frequently identified replicon was rep10, which was detected in 47% (*n* = 7/15) of the isolates. A combination of rep20/rep5a (40%, *n* = 6/15) was also frequently detected, followed by rep16/rep5a (20%, *n* = 3/15). RepL was the most identified replicase and was associated with a replicon 10, whereas the rep16/rep5a and rep20/rep5a were also more frequently associated with Inc18/Rep3 and RepA‐N/Rep3, respectively. Nine isolates carried plasmids encoding either *blaZ* and/or *ermC* resistance genes.

**Table 4:** Distribution of plasmid replicons and carried AMR genes in MRSA isolates.

Site	Isolates	ST	Replicon	Replicase domain	Coverage (%)	AMR gene
Facility 1	SA2148	1	rep16/rep5a	Inc18/Rep3	100	PC1‐*blaZ*
			rep7c	Rep_trans	100	None detected
	SA2285	22	rep10	Repl	100	*erm*(C)
	SA2443	22	rep10	RepL	100	*erm*(C)
	SA2467	22	rep10	RepL	100	*erm*(C)
	SAA519	22	rep10	RepL	100	*erm*(C)
	SAA238	78	rep16/rep5a	Inc/Rep3	100	PC1‐*blaZ*
	SA5943	78	rep16/rep5a	Inc18/Rep3	100	PC1‐*blaZ*
	SA2415	6963	rep20/rep5a	RepA‐N/Rep3	100	None detected
	SAA237	6963	rep20/rep5a	RepA‐N/Rep3	100	None detected
	SAA035	6963	rep10	RepL	100	*erm*(C)
			rep20/rep5a	RepA_N/Rep3	100	None detected

Facility 2	SA2481	45	rep10	RepL	100	*erm*(C)
			rep21/rep20	Rep_1/RepA_N	100/67.01	PC1‐*blaZ*
			rep7a	Rep_trans	100	*tet*(K)

Facility 3	SA3499	22	rep20/rep5a	RepA‐N/Rep3	100	None detected
	SA6221	22	rep20/rep5a	RepA‐N/Rep3	100	None detected
	SAA005	22	rep20/rep5a	RepA‐N/Rep3	100	None detected
	SAA254	22	rep10	RepL	100	*erm*(C)
			rep20	RepA‐N	100	None detected
			rep5a	Rep3	100	None detected

### 3.12. SNP Analysis Detected Clonality and Potential Transmission of Isolates

A core genome SNPs analysis was used to assess clonality among isolates. The construction of a core‐genome phylogenetic tree of the isolated MRSA revealed clustering of isolates. According to previous studies, isolates showing less than 50 SNPs were considered as genetically related clones [58, 59].

Two isolates (SA2415 and SAA237), with less than 46 SNPs between them were recovered from one resident (Resident 1) over two separate collection periods, indicating colonization (Figure 8). Isolate SAA035 was isolated from the nasal swab of Resident 2 and differed by 46 SNPs when compared with the isolates from Resident 1. Both residents live in the same facility indicating that this clone is circulating in the facility. Genetically related isolates SA2285, SA2443, and SAA519 were isolated from the nasal swab, urine‐, and wound samples, respectively, of the same resident (Resident 3), whereas the related isolate SA2467 was recovered from a urine sample of another resident (Resident 4) in the same facility (Figure 8). Resident 4 did not seem to carry MRSA as two nasal swabs from this resident, one on a date before and one on a date after the positive urine sampling, did not yield any MRSA. Therefore, this could possibly indicate transmission of this clone between residents.

**Figure 8:**
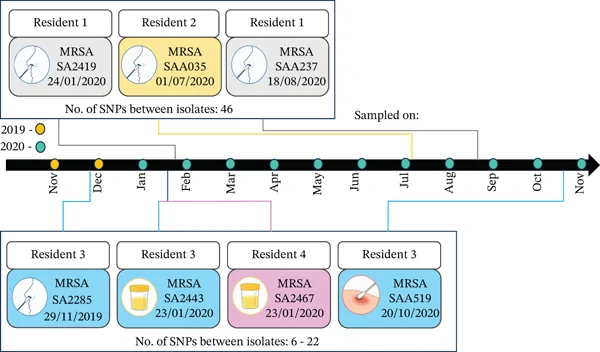
Colonization, circulation, and possible transmission of MRSA isolates between residents in the same facility.

Persistent colonization/infection by clonal MRSA (SNP less than 49) was also observed in three other residents (Residents 5, 6, and 7) at different sampling times ranging from 4 to 6 months (Figure 9).

**Figure 9:**
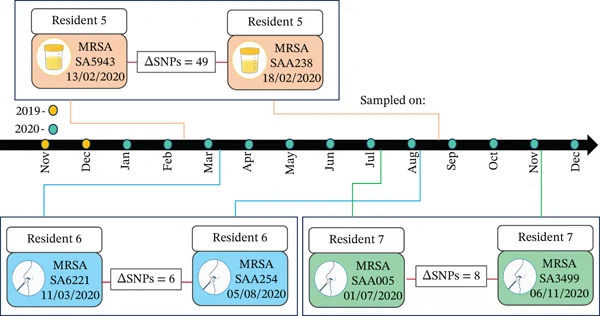
Persistent colonization of MRSA isolates between residents in the same facility.

## 4. Discussion

In this study, MRSA colonization was identified in 8.2% of residents across three RACFs, a prevalence comparable with the 10% reported in South Australia more than two decades earlier [60]. The similarity in prevalence despite the 22‐year interval may suggest that MRSA carriage within South Australian RACFs has remained relatively stable over time, potentially reflecting effective infection prevention and antimicrobial stewardship practices that have prevented substantial increases in transmission. However, direct comparisons should be interpreted cautiously, as differences in study design, sampling methods, facility characteristics, and resident demographics may influence prevalence estimates. Carriage is also similar to that reported in Japan (9.8%) [61], but lower than the 16% reported in Melbourne [9]. Rates vary globally, with lower prevalence in Brazil (3.7%) [62], Germany (5.5%) [63], and Luxembourg (7.2%) [64]. Such variation is likely influenced by differences in infection control policies, healthcare system structures, screening practices, antimicrobial resistance burden, and the prevalence of MRSA within surrounding healthcare networks.

All isolates were strong biofilm producers, reinforcing the importance of biofilm formation as a mechanism facilitating persistent colonization in RACF residents. The universal biofilm‐forming capacity observed among isolates may partly explain the persistence of MRSA colonization identified in some residents and its ongoing circulation within facilities.

Although 66.6% of isolates were MDR, this proportion was lower than that reported in RACFs in Croatia (75.5%) [65] and Greece (97%) [66]. Nevertheless, the MDR rate remains concerning, as it substantially reduces treatment options and may increase the risk of adverse outcomes in older populations, who often have multiple comorbidities and frequent healthcare exposure. The coexistence of MDR and strong biofilm production may further complicate eradication efforts because biofilms can enhance bacterial tolerance to antimicrobial agents beyond that conferred by conventional resistance mechanisms.

The inability to identify known resistance determinants for doxycycline, tetracycline, and tigecycline resistance may indicate the presence of uncharacterized resistance mechanisms or mutations not captured in current resistance databases. This finding highlights the ongoing need to expand resistance gene databases and investigate emerging resistance mechanisms in circulating MRSA populations.

The predominance of ST22 is consistent with reports demonstrating the successful global expansion of epidemic methicillin‐resistant *S*. *aureus*‐15 (EMRSA‐15) [67–69] and its establishment as a dominant healthcare‐associated lineage in Australia [70]. The predominance of a single clone within multiple RACFs suggests that transmission may be driven not only by resident‐to‐resident contact but also by shared healthcare networks, including hospitals, outpatient services, and staff movement between facilities. ST22 is associated with SCC*mec* IV [71]. Type IV SCC*mec* is smaller than other SCC*mec* types as it lacks much of the additional resistance cargo present in larger SCC*mec* elements but imposes a lower fitness burden on the bacterium. This characteristic may enhance transmissibility and ecological fitness, facilitating dissemination across healthcare settings [72]. The detection of ST6963 is particularly noteworthy because it provides evidence of ongoing microevolution within the dominant ST22 lineage. The close genetic relationship between ST6963 and ST22 [73] suggests recent divergence, [73] potentially reflecting local adaptation within Australian healthcare or aged‐care environments. The emergence of novel STs within RACFs may be facilitated by prolonged colonization, recurrent antibiotic exposure, and close‐contact transmission networks that create opportunities for bacterial evolution and persistence. Genomic analysis of SA2481 (ST45) revealed the presence of a complex composed of cassette chromosome recombinases, the ccrC1 region, and internal mobile elements (IS431), but an absence of the *mecA* gene. Despite lacking the *mecA* gene, SA2481 (ST45) exhibited low‐level resistance to cefoxitin. A distinct lineage of multidrug‐resistant ST45 MRSA harboring the drug efflux pump, QacA, is emerging predominantly in Australia and Singapore [19].

Interestingly, ST45, ST78‐IV, and ST1‐IV were classified as healthcare‐associated MRSA despite belonging to lineages more commonly associated with community acquisition [70, 74–76]. This observation suggests a blurring of the traditional distinction between community‐ and healthcare‐associated MRSA. Increasing movement of residents between hospitals, RACFs, rehabilitation facilities, and the community creates opportunities for bidirectional transmission, resulting in the establishment of traditionally community‐associated clones within healthcare settings.

SNP analysis confirmed persistent colonization and circulation of clones within facilities, including one resident harboring clonally related MRSA isolates in the nose, wound, and urine. This resident received 11 courses of antibiotics during the year of sampling. The concomitant observation of clonal transmission of MDR *Klebsiella pneumoniae* within the same RACFs [10] reflects broader infection prevention and control challenges within the aged‐care environment.

This study provides insights into MRSA prevalence, resistance, and transmission in RACFs. The observation that nasal carriage corresponded colonization and clonal circulation suggests the use of nasal screening as a practical surveillance tool. As all the isolates were susceptible to mupirocin, targeted decolonization strategies, including intranasal decontamination protocols, may therefore represent a useful intervention for reducing MRSA colonization, transmission, and subsequent infection in RACF populations [77].

**Nomenclature**
ATCC: American Type Culture Collection
Ccr: cassette chromosome recombinase
CFU: colony‐forming unit
EMRSA: epidemic methicillin‐resistant *Staphylococcus aureus*
EUCAST: European Committee on Antimicrobial Susceptibility Testing
MALDI‐TOF MS: matrix‐assisted laser desorption/ionization‐time of flight mass spectrometry
MDR: multidrug‐resistant
MIC: minimum inhibitory concentration
MRSA: methicillin‐resistant *Staphylococcus aureus*
OD: optical density
PBP: penicillin binding protein
PVL: Panton–Valentine leukocidin
RACFs: residential aged care facilities
SCCmec: staphylococcal cassette chromosome *mec*
SNP: single nucleotide polymorphism
Spa: S*taphylococcus aureus* protein A (spa gene)
ST: sequence type
UTI: urinary tract infection

## Funding

This study was supported by the Australian Medical Research Future Fund (GN1152556).

## Disclosure

The collective WGSs of isolates used in this study were deposited to the NCBI database BioProject “PRJNA1122970.”

## Ethics Statement

All participants provided their informed consent to participate as volunteers. No incentives were offered. The project was approved by the Human Research Ethics Committee of the University of South Australia (Application ID: 201882).

## Conflicts of Interest

The authors declare no conflicts of interest.

## Data Availability

The data that support the findings of this study are openly available in NCBI at https://www.ncbi.nlm.nih.gov/home/genomes/, Reference Number PRJNA1122970.
